# Cheerfulness

**Published:** 1886-12-11

**Authors:** 


### WORDS OF CONSOLATION.

[Communications for this column should be addressed, "Chaplain," care of the Editor of THE HOSPITAL.]

### XI.—CHEERFULNESS.

WE have no scruple about devoting another article to the subject of Cheerfulness; so important do we consider the exercise of this gift, both for the sake of patients and attendants. Cheerfulness in a patient not only causes the mind to act beneficially upon the body, and so becomes a sort of handmaid to the physician's remedies; but it also has a reflex action upon the nurses, and makes it much easier for them to perform their arduous duties. In this way it may be said to produce its own reward. For, although a nurse may try and be as impartial as possible, and make a point of treating all her sufferers equally well, it is but human nature to incline more favourably to those who are evidently striving to be cheerful. In ordinary life, we are most of us naturally drawn and interested by cheerful companions. We like to have such people about us. We see, too, that because their fellows gravitate towards them they make much happiness for themselves. Cheerfulness is much more difficult for the sick and suffering to maintain. Nevertheless, what is true under average circumstances holds especially good in the case of sufferers. If they maintain cheerfulness they attract the sympathy of those who minister unto them; and by the force of this attraction they procure, shall we not say, the best of their nurse's attention, and the very essence of hospital watchfulness. We repeat, a nurse may be most impartial, yet she cannot but feel. "It is easy enough to attend to this patient, he is so cheerful. There is not half so much pleasure in attending to that one, he is so desponding or discontented." All our ministrations to one another vary, often imperceptibly to ourselves, according to the spirit in which they are received. The cheerful make to themselves friends. Nowhere is this truth more completely illustrated than in a hospital.

Again, these very friends once made, who find it such a pleasure to minister to the happier minded patients, have their own mental and spiritual energies immensely strengthened thereby. The mental strain which the attendants at lunatic asylums undergo in the effort to maintain constant cheerfulness is, we believe, very great, because they get so little encouragement from the poor imbeciles and people suffering from melancholy madness. The strain in a hospital is, of course, not so severe; but it must always be in proportion to the dispositions of the patients. Therefore let each one be sure that cheerfulness has much to to do with his or her own recovery. Let each one believe that it has a decided influence upon the conduct and care of the nurses. Let each one consider, as well, that when he entered the hospital he came in not merely as an individual to get what good he could out of it, but rather as a member of a community whose bounden duty it is to try and promote the general efficiency of the institution. How can the general efficiency be better promoted than when all concerned in suffering and nursing, are bent on creating and sustaining an atmosphere of cheerfulness? "God loveth a cheerful giver" (2 Cor. ix, 7). Must he not also love a cheerful sufferer.

*(To be continued.)*

